# Frontline Staff Experiences Delivering Home Health in a Pilot Veterans Health Administration Program

**DOI:** 10.1093/geroni/igaf122.319

**Published:** 2025-12-31

**Authors:** Heather Davila, Mary Good, Tammy Walkner, Heather Benzel, Irene San Roman, Aaron Seaman

**Affiliations:** Iowa City VA Healthcare System, Iowa City, Iowa, United States; Iowa City VA Health Care System, Iowa City, Iowa, United States; Iowa City VA Health Care System, Iowa City, Iowa, United States; VA Midwest Healthcare Network, St. Paul, Nebraska, United States; Iowa City VA Health Care System, Iowa City, Iowa, United States; University of Iowa, University of Iowa, Iowa, United States

## Abstract

Historically, the Veterans Health Administration (VHA/VA) has provided access to skilled home health (e.g., rehabilitation, nursing) and home health aide services through contract community agencies. In 2022, VA began piloting VA-delivered home health (VA-HH) at 4 sites in the upper Midwest. Program goals were to improve access to care and care coordination. In 2024, we conducted semi-structured interviews with VA-HH nurses (n = 18) and nursing assistant/aides (n = 13) to understand motivations for joining VA-HH, resources needed to address Veterans’ complex care needs, and overall job satisfaction. Most nurses (60%) and aides (85%) joined VA for their VA-HH positions. Motivations included commitment to the philosophy of home health, desire to give back to Veterans, and appreciation for flexibility and autonomy in their roles. Both groups noted the need for clear guidelines and procedures, supplies (e.g., for wound care), and technology (e.g., for in-home charting) to effectively deliver home health to Veterans with complex care needs. Whereas nurses discussed the importance of inter-team collaboration (e.g., with social work, primary care), aides focused more on collaboration within the VA-HH team. Aides described needing strong communication with nurses, plus nurse and management support related to complex care (e.g., dementia, mental health) and safety concerns. Most staff expressed high job satisfaction. Aides, particularly those with prior non-VA experience, noted being valued and included in the overall healthcare team. Moving forward, VA-HH could be expanded to serve more Veterans. Further enhancing inter- and intra-team collaboration, safety protocols, and leadership support could promote ongoing job satisfaction and high-quality care.

